# Effect of Hydrophilic Polymers on the Release Rate and Pharmacokinetics of Acyclovir Tablets Obtained by Wet Granulation: In Vitro and In Vivo Assays

**DOI:** 10.3390/molecules27196490

**Published:** 2022-10-01

**Authors:** D. Nagasamy Venkatesh, Subramanianainar N. Meyyanathan, Andjelka Kovacevic, Aleksandra Zielińska, Joel Fonseca, Piotr Eder, Agnieszka Dobrowolska, Eliana B. Souto

**Affiliations:** 1 Department of Pharmaceutics, JSS College of Pharmacy, JSS Academy of Higher Education & Research, Rocklands, Post Box. No. 20, Elk Hill Road, The Nilgiris, Ooty 643001, Tamil Nadu, India; 2 Department of Pharmaceutical Analysis, JSS College of Pharmacy, JSS Academy of Higher Education & Research, Rocklands, Post Box. No. 20, Elk Hill Road, The Nilgiris, Ooty 643001, Tamil Nadu, India; 3 Department of Pharmaceutical Technology, Institute of Pharmacy, Friedrich-Schiller University Jena, Helmholtzweg 4, 07743 Jena, Germany; 4 Institute of Human Genetics, Polish Academy of Sciences, Strzeszyńska 32, 60-479 Poznan, Poland; 5 Department of Pharmaceutical Technology, Faculty of Pharmacy, University of Porto, Rua Jorge de Viterbo Ferreira, 228, 4050-313 Porto, Portugal; 6 Department of Gastroenterology, Dietetics and Internal Diseases, Poznan University of Medical Sciences, 60-355 Poznan, Poland; 7 REQUIMTE/UCIBIO, Faculty of Pharmacy, University of Porto, Rua Jorge de Viterbo Ferreira, 228, 4050-313 Porto, Portugal

**Keywords:** acyclovir, hydrophilic polymers, sustained release profile, pharmacokinetics, in vitro and in vivo testing

## Abstract

This study aims to evaluate the feasibility of producing acyclovir-containing modified release matrix tablets by a wet granulation method based on the type and concentration of two pharmaceutical-grade hydrophilic matrix polymers (i.e., hydroxypropyl methylcellulose (HPMC), carbomers, and their combinations) commonly used in biomedical applications. The mechanical properties of the tablets and in vitro and in vivo performance were studied. The physicochemical properties of the raw materials and corresponding physical mixtures were characterized by differential scanning calorimetry, showing that the hydrophilic polymers did not influence the physicochemical properties of the drug. The wet granulation process improved the flow and compression properties of the obtained granules. This method enabled the preparation of the matrix tablets of acyclovir with appropriate mechanical properties concerning hardness and friability. The drug release kinetics was governed by the type and concentration of the hydrophilic polymers composing the matrices. The study has proven that HPMC-composed tablets were superior in modified drug release properties compared to carbomer- and HPMC/carbomer-based tablets. Mathematical analysis of the release profiles, determined in a medium adjusted to pH 1.2 followed by pH 7.4, revealed that the drug released from the hydrophilic tablets followed non-Fickian first-order kinetics. An optimal HPMC-based formulation submitted to accelerated stability studies (40 °C, 75% RH) was stable for three months. A complete cross-over bioavailability study of the selected acyclovir-loaded sustained release tablets and marketed immediate-release tablets were compared in six healthy male volunteers. The extent of drug absorption from the sustained release tablets was significantly greater than that from immediate-release pills, which may improve the drug’s antiviral properties attributed to the lower elimination rate and enhanced acyclovir half-life.

## 1. Introduction

Acyclovir (ACY) is a well-known antiviral drug that inhibits the activity of human herpes viruses both in vitro and in vivo, including herpes simplex virus type 1 (HSV-1, or oral herpes virus) and type 2 (HSV-2, or genital herpes virus), which cause herpes keratitis, herpes simplex encephalitis, genital herpes, neonatal herpes, and herpes labialis) and varicella zoster virus (chickenpox, herpes zoster, or shingles virus) [1]. According to the Biopharmaceutical Classification System (BCS), ACY is a class three drug (high solubility and low permeability) when administered as immediate-release tablets up to 400 mg. It can also be classified as a class four drug (low solubility and low permeability) when the amount of the immediate release tablet is 800 mg [2,3,4]. This drug must be administered frequently (usually five times per day, a tablet of 200 mg (1000 mg daily) for genital herpes, or four tablets of the same dose (4000 mg daily) for herpes zoster treatment) due to low absorption (about 20%) and short t_½_ (three hours) [5,6]. Frequent administration and high drug dosage may compromise patient compliance. Only a few studies document an interest in designing sustained-release (SR) systems to achieve optimal therapy with drugs that have a narrow therapeutic window or are eliminated rapidly to maintain effective systemic drug concentrations for long periods. SR systems are designed to immediately bring the blood level of a drug to its therapeutic attention through an initial dose portion and then sustain this level for a certain predetermined period with the maintenance dose within the therapeutic window [7,8]. This approach means the reduction of the administered doses and lower overdose incidence. It may represent a promising way to treat patients with herpes simplex and varicella zoster viruses. Modulating/sustaining the drug release is a method that includes the drug in a matrix, which can swell to form a continuous gel layer that controls drug release. Few investigations report hydrophilic matrix-controlled release dosage forms for oral delivery of ACY [9,10,11]. Hydrophilic polymeric matrix systems are widely used in oral sustained/controlled drug delivery due to the flexibility in obtaining the desirable drug release profile [12]. Hydration of polymer causes the formation of a gel layer that controls the release rate of the drug. In contrast, in vitro drug release of water-soluble drugs is controlled by diffusion out of the gel layer, i.e., depends on the gel viscosity. On the other hand, the release of poorly water-soluble medicines depends solely on polymer dissolution [13,14].

Cellulose derivatives have been widely used to formulate hydrogel matrices for controlled drug delivery. Hydroxypropyl methylcellulose (HPMC) is the most extensively employed because it is easy to use, available, and has very low/no toxicity [15]. In this case, drug release is controlled by the hydration of HPMC. A gelatinous layer at the matrix surface, through which the loaded drug diffuses, is created [16].

In matrix tablets prepared with HPMC, a gelatinous layer is formed on the surface of the tablets upon hydration. At the higher concentrations, the linear polymer chains entangle to a greater degree, resulting in ‘virtual crosslinking’ and forming a more robust gel layer. Upon dissolution, this gel layer erodes, resulting in drug release. In addition, this polymer’s high molecular weight and crosslinked acrylic polymers (carbomers) also offer a variety of desirable features for controlled drug release. In contrast to HPMC, the drug is entrapped in the glassy core in the dry state of tablets formulated with carbomer. As the external surface of the tablet is hydrated, it also forms a gelatinous layer upon hydration. However, this gel layer is structurally different from HPMC matrix tablets. In carbomer-based tablets, when the hydrogel is fully hydrated, osmotic pressure generated inside the matrix breaks up the structure by sloughing off discrete pieces of the hydrogel. These hydrogels remain intact, and the drug diffuses through the gel layer uniformly.

This study performed the feasibility of producing compressed matrix tablets of ACY based on HPMC, Carbopol, or their combinations as hydrophilic polymeric matrices. A polyvinylpyrrolidone solution in isopropyl alcohol as a granulation fluid was used in this case. The matrix tablets were produced by the conventional wet granulation method. The effect of these polymers and their concentrations on the in vitro and in vivo drug release rate was studied for the most promising formulation concerning modified release.

## 2. Results and Discussion

**Figure 1:**
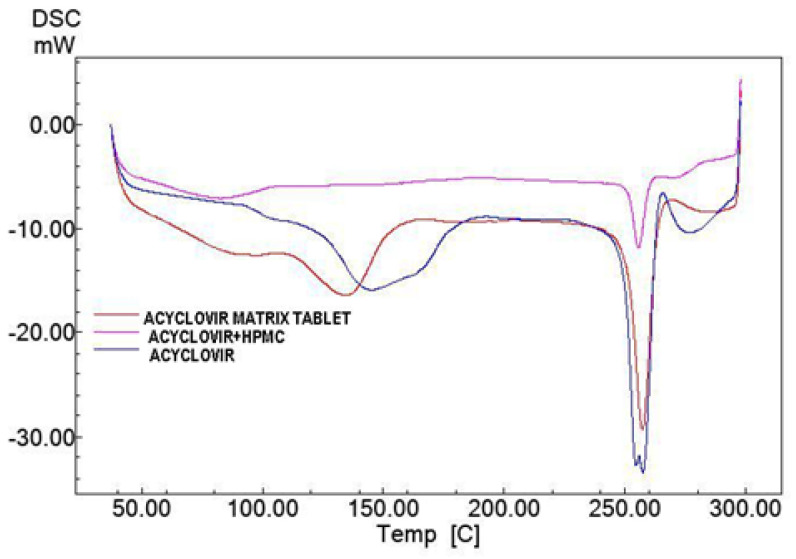
DSC thermograms of acyclovir, polymer, and acyclovir matrix.

The thermograms of ACY, the physical mixture of ACY and HPMC (1:1 ratio *w*/*w*), and ACY matrix tablets are shown in Figure 1. According to the literature, the melting point of acyclovir is 257 °C [17], which is very close to the endothermic peak seen in the DSC analysis of the drug alone, i.e., at 256 °C. In contrast, the pure HPMC showed its endothermic peak at 87.91 °C. The overlaps in the recorded profiles for the drug alone, the drug combined with HPMC, and the final tablets confirm no chemical interaction between ACY and the excipients used in the tablet composition.

The manufacturing process of tablets follows a complex pathway to render the components of the formulation (drug and excipients) compression, while keeping flowability of powders during production and disintegration of tablets and dissolution of the drug upon oral administration. The success of mixing and demixing powders is governed by their particle size and shape, surface electrostatic charge, hydrophilic properties, and polymorphism. For an optimal mixing time, a suitable powder mixture is ensured when the different powders to be mixed are of similar mean particle size, as this will contribute to the drug being uniformly distributed within the excipients and reduce the risk of powders segregation in the tablet machine. If segregation happens, problems of mass uniformity, hardness, and content uniformity will occur in the produced tablets. The first step is thus to reach a mean range of particle size of powders before adding lubricants, and this is accomplished by mesh calibration, which was performed for HPMC and Carbopol using a sieve of 0.25 mm diameter. Following this step, wet granulation is followed by adding a solution of PVP K-30 in isopropyl alcohol as a granulation medium to the powder mixture. PVP or povidone in solution is a standard binder in wet-granulation processes. It has been shown to improve the dissolution of poorly water-soluble drugs in oral solid dosage forms. It is regarded as a biologically inert and non-toxic excipient [18]. For granule production, the obtained wet mass was passed through a sieve of 1.7 mm diameter using a stainless-steel spatula at room temperature, which was then dried at 60 °C over three to four hours until a drying loss lower than 2% (*w*/*w*) was achieved. Microcrystalline cellulose (Avicel PH 101), magnesium stearate, and talc were added to the dried granules. Magnesium stearate was used to reduce surface electrical charges acting as a lubricant and talc to improve the dried granules’ flowability. Microcrystalline cellulose acts as a diluent. The flowability properties were evaluated by determining the angle of repose, LBD, TBD, Carr’s compressibility index, and HF [19]. The ACY powder was too cohesive to flow through the funnel. Thus, no angle of repose could be recorded. Still, the calculated HF (the ratio between the tapped bulk density and the loose bulk density) was 2.18, which translates to the cohesiveness of acyclovir powder and, thus, deficient flowability properties [20]. The dried granules, on the other hand, showed improved flowability as seen by the angle of repose recorded between 21.99 ± 0.05° and 23.70 ± 0.06° and the HF values between 1.15 and 1.35, resulting from the reduction of interparticle friction, lowering the tapped bulked density, and increased particle size compared to non-granulated powder (Table 1).

Carr’s index refers to the percentage compressibility and is an indirect method to evaluate the powder flowability from bulk densities [21]. The compressibility of ACY powder was found to be 54.05%. This result supports the conclusion that granulation improved flowability and compressibility, as confirmed by the values of angle of repose and HF. The hydrophilic polymers’ type, concentration, and ratio did not influence the physical properties of the obtained granules.

Table 2 summarizes the results of the physical characterization of the tablets. The standard deviation of the mass uniformity measurements was very uniform, ranging from 0.004 to 0.012. According to the European Pharmacopoeia, for uncoated and film-coated tablets with an average mass of 250 mg or more, the accepted percentage deviation is 5% [22]. In our case, the mass variation was below 3%. The variation of tablets’ thickness was also found to be minimal, i.e., between 5.21 ± 0.14 mm and 5.30 ± 0.08 mm. The increase of the polymer concentrations resulted in no alteration of tablets’ thickness, which may translate that polymer does not alter the binding properties.

Concerning friability, European Pharmacopoeia says that for a single test or the mean of three tests, the maximum loss of mass cannot be more significant than 1% to be considered acceptable. From data shown in Table 2, we see no marked differences in the friability (ranging between 0.41 ± 0.01 and 0.49 ± 0.001%) obtained with the tablets prepared using different polymer concentrations. The hardness of the prepared tablets fell into the range of 5.16 ± 0.22 to 5.82 ± 0.47 kg/cm^2^. Regardless of the polymer type, the increase in its concentration does not significantly change the hardness of the tablet (*p* > 0.05). These results are in agreement with the outputs seen for thickness and friability. 

Tablets with mass and content uniformity are also the first step to reach uniformity in the release profile within the same batch of tablets. In addition, the assay has to be run at different pH media to ensure a sustained release.

**Figure 2:**
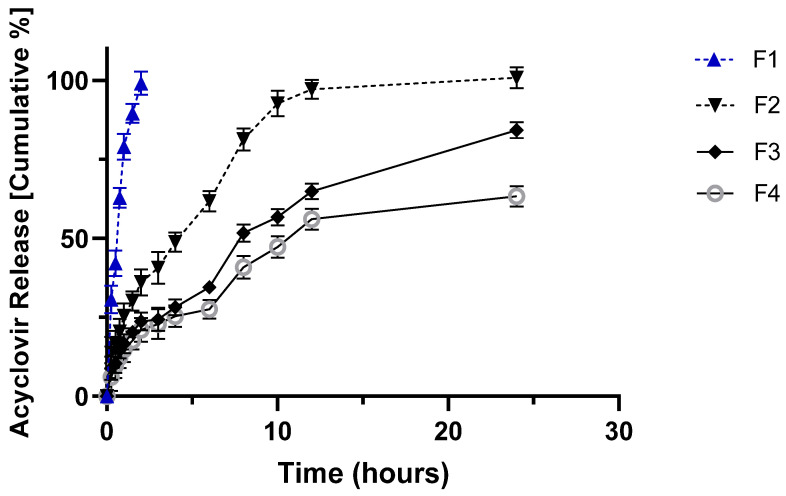
In vitro release profile of acyclovir from HPMC tablets (F-1 to F-4) in 0.1 N HCl (pH 1.2) from zero to two hours, after which 200 mL of 0.2 M sodium phosphate tribasic solution was added to reach a final pH of 7.4 kept for the remaining 24 h. Please refer to Table 7 for tablets’ composition.

**Figure 3:**
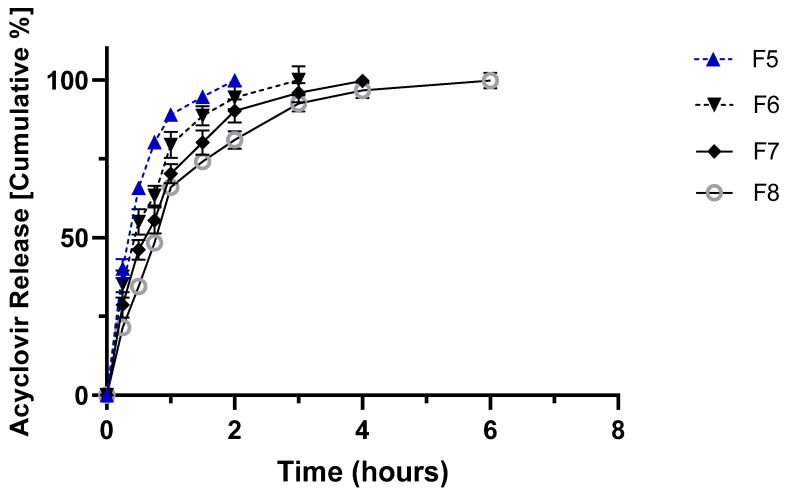
In vitro release profile of acyclovir from Carbopol tablets (F-5 to F-8) in 0.1 N HCl (pH 1.2) from zero to two hours, after which 200 mL of 0.2 M sodium phosphate tribasic solution was added to reach a final pH of 7.4 kept for the remaining 24 h. Please refer to Table 7 for tablets’ composition.

**Figure 4:**
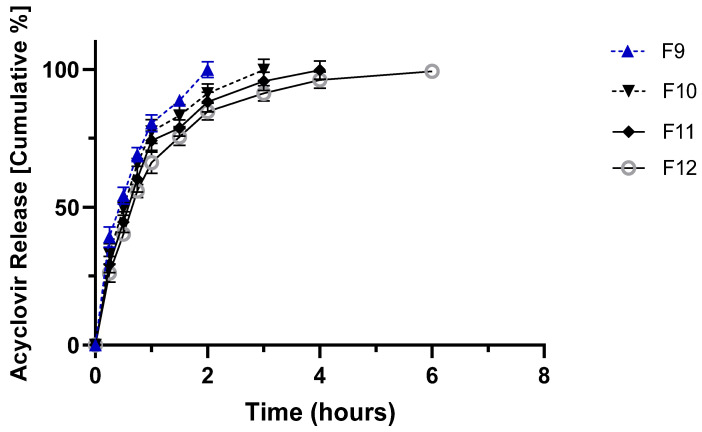
In vitro release profile of acyclovir from HPMC and Carbopol tablets (F-9 to F-12) in 0.1 N HCl (pH 1.2) from zero to two hours, after which 200 mL of 0.2 M sodium phosphate tribasic solution was added to reach a final pH of 7.4 kept for the remaining 24 h. Please refer to Table 7 for tablets’ composition.

Figure 2, Figure 3 and Figure 4 show the in vitro release profile at pH 1.2 and 7.4 of ACY from the prepared hydrophilic matrix tablets for 24 h.

The drug release profile was found to be sustained for all formulations developed according to the composition shown in Table 7 (see Materials and Methods). Interestingly, the increased concentration of HPMC resulted in a decrease in release rate, whereas Carbopol matrices released ~80% of the drug within two hours, regardless of the polymer concentration (Figure 3). Combining Carbopol with HPMC also promoted a burst release of more than 80% of the drug within the first three hours (Figure 4). This result was already seen in preliminary work using prochlorperazine maleate in modified release tablets [23]. HPMC can be used as a binder and as a matrix for modified-release tablets. Although soluble, in cold water HPMC forms a viscous solution; HPMC is practically insoluble in hot water. In the release medium set at 25 °C, HPMC forms a diffusion layer that controls the release of ACY from the matrix.

On the other hand, the presence of the acrylic acid-based polymer in the matrices induced a significant burst release of ACY attributed to the disintegration properties of Carbopol [24]. The combination of Carbopol and HPMC showed moderate sustained release (Figure 4) compared to HPMC alone (Figure 2). The identified differences among the release profiles from the different polymer compositions cannot be attributed to the mechanical/physical properties of the tablets, as no significant differences were recorded among the data depicted in Table 2.

On the other hand, wet granulation is another processing factor that may play a relevant role in the tablet’s profile in vitro. Firstly, it is essential to control the rate of addition and concentration/volume of the granulation fluid as the drug may dissolve or recrystallize in a different form and compromise the release rate. Secondly, the size and the type of granules present in the mixture may also influence the porosity of the tablets and thus their disintegration and dissolution. As the porosity affects the tablets’ wettability, the loss on drying (LOD) is also relevant to ensure that granules are dried enough that relatively low moisture is obtained. Usually, LOD is between 1% and 3%.

Although the release kinetic studies were performed in perfect sink conditions, a significant amount of ACY was released under acidic pH (in 0.1 N HCl, pH 1.2) compared to that terminated in a more alkaline medium (phosphate buffer, pH 7.4). From these outcomes, we can anticipate that ACY’s in vitro release profile strongly depends on the drug’s solubility in the dissolution medium.

Mathematical models were used to calculate the regression coefficient and anticipate the best fit to describe the release kinetics of ACY from the developed tablets. In all formulations, the highest fit with a more significant correlation coefficient (r^2^ > 0.98) was found for Higuchi’s model for [25], typically described using the following Equations (1) and (2) [26]:

(1) $$M_{t}=\sqrt{DC_{s}\left(2A-C_{s}\right)t}$$

or

(2) J=dMtdt=DCs(A−12Cs)2t

where *M_t_* stands for the cumulative amount of drug being released up to time *t* as a function of unit surface area, and *D* for the coefficient of diffusion of the drug in the polymeric matrix, this model describes the release rate of a drug from a polymeric matrix where the concentration of drug *A* exceeds its solubility *C_s_* in the matrix into the releasing medium. The Higuchi model, however, is not ideal for evaluating the effect of tablet hydration and swelling and the impact of matrix erosion. Thereupon, the dissolution data were fitted to the following Equation (3) [27]:

(3) $$\frac{M_{t}}{M_{\alpha }}=k.t^{n}$$

*M_t_/M_α_* describes the fraction of drug release at the time *t*, and *k* is the kinetic constant; *n* is the release exponent, indicating the drug release mechanism. In addition, to determine *n*, only the initial portion of the release curve (*M_t_/M_α_* < 0.6) is used [28,29]. Ritger and Peppas [30] described the exponent *n* as a function of 2*a/L*, which is the ratio of diameter (2*a*) and thickness (*L*) of the solid dosage form. It is represented by the slope of the log fraction of ACY release versus the log time curve. In the case of tablets, if the exponent *n* values fall within 0.43 and 0.5, this indicates a Fickian diffusion mechanism, i.e., Case I release kinetic. If exponent *n* values fall between 0.5 and 0.89, a non-Fickian diffusion mechanism or anomalous release kinetics is described (e.g., in the case of polymer relaxation). If the exponent *n* is 1, it is zero order kinetics, whereas a super case II happens when the exponent *n* is above 0.89. To determine the mean dissolution time (MDT), the following Equation (4) was used:

(4) MDT=nn+1×k−1n

where *n* is the release component and *k* is the kinetic constant. Table 5 summarizes the values of the exponent *n*, correlation coefficients r^2^, and MDT, together with the zero order, first order, and Higuchi models. All formulations’ correlation coefficients (r^2^) were high enough to predict the drug dissolution behavior (r^2^ = 0.9538–0.9929) for any of the models. Regardless of the concentration of polymer, a non-Fickian release profile with an exponent *n* between 0.5 and 0.89, was seen.

**Figure 5:**
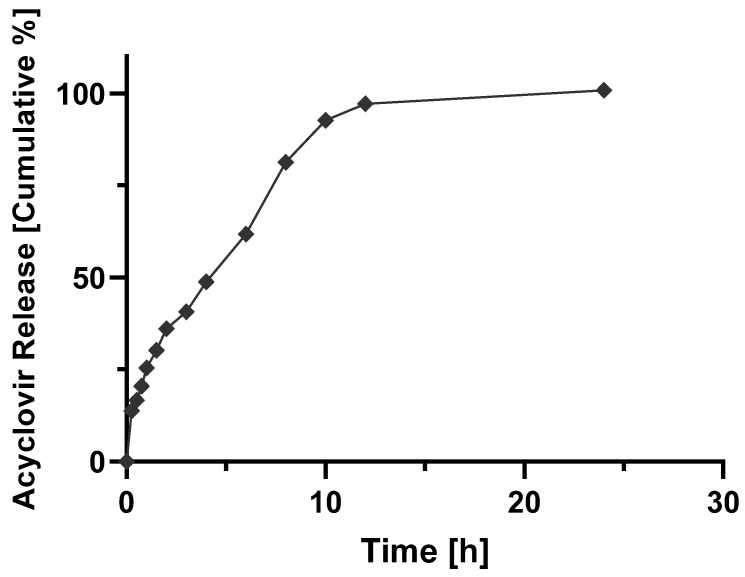
Cumulative percentage of acyclovir from F2 released formulation as a function of time (h) fitted to zero order kinetics ($y=4.452x+24.332;\;R^{2}=0.7703$).

**Figure 6:**
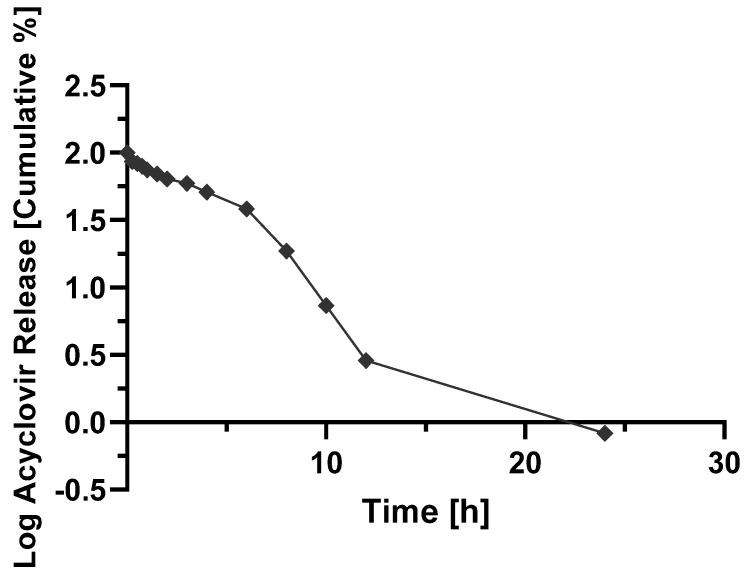
Logarithm cumulative percentage of acyclovir from F2 released formulation as a function of time (h) fitted to first order kinetics ($y=-0.1134x+2.0381;\;R^{2}=0.940$).

**Figure 7:**
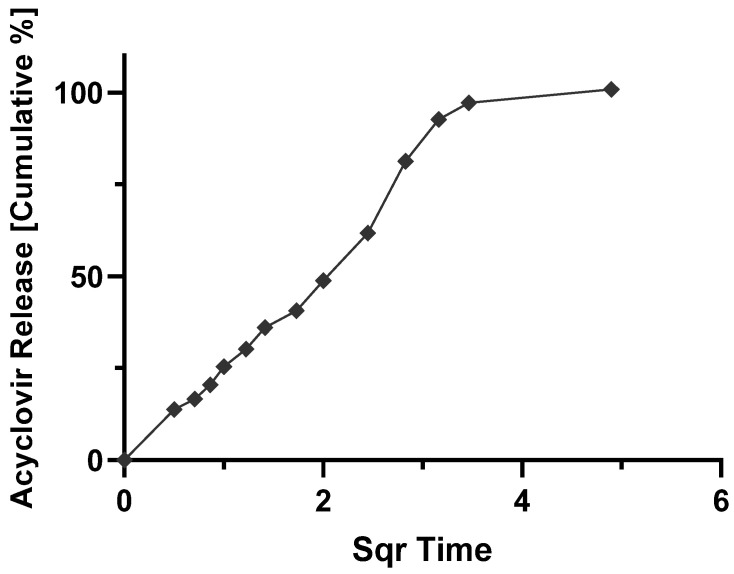
Cumulative percentage of acyclovir released from F2 formulation as a function of square route of time fitted to Higuchi kinetics ($y=24.137x+2.2932;\;R^{2}=0.9412$).

**Figure 8:**
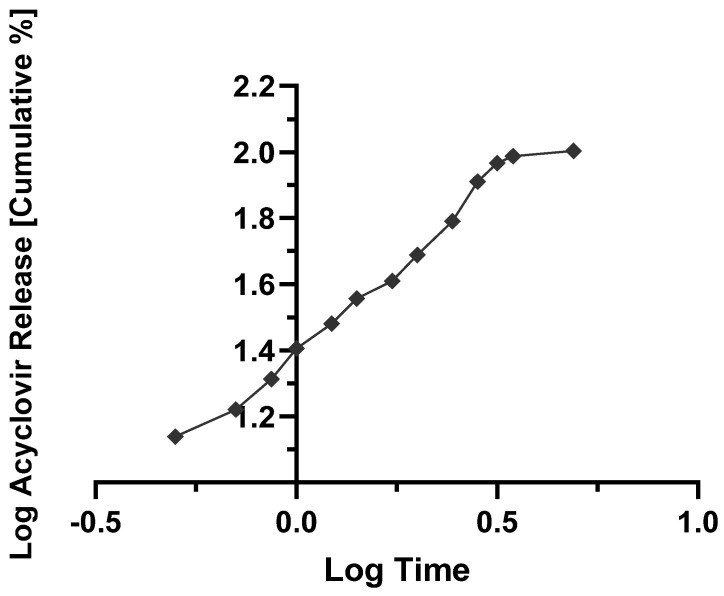
Logarithm of cumulative percentage of acyclovir released from F2 formulation as a function of logarithm of time fitted to Peppas kinetics ($y=0.5011x+1.4022;\;R^{2}=0.9799$).

The contact hydrophilic tablets, as those produced by wet granulation in this work, get in contact with the dissolution medium, firstly at pH 1.2 and two hours later at pH 7.4, an initial (burst) release is seen from the drug particles placed more on the surface of the tablets. The polymer relaxation follows this as the result of the swelling of the solid matrix; the drug starts dissolving in the liquid that penetrates/permeates the solid dosage form, and the generated viscous polymer layer controls the release profile of the drug into the outer dissolution medium. In parallel with the diffusion of the dissolved drug, erosion of the viscous polymer layer occurs, generating aggregates or granules, which are further disintegrated into finer particles. For a better illustration, Figure 5, Figure 6, Figure 7 and Figure 8 show the mathematical modeling profiles of F2 fitted to zero order (Figure 5), first order (Figure 6), Higuchi (Figure 7), and Peppas (Figure 8) models. Based on the improved modified release profile seen for HPMC matrices, F2 was selected. It took 12 h to release 100% of ACY in the dissolution medium, whereas F1 took less than three hours to release the total amount of formulated drug, and F3 and F4 did not reach 90% by the end of the release assay.

The MDT can be used to evaluate the retarding-efficiency effect of the polymer composing the modified release tablets and to characterize the drug release rate [31]. The higher the MDT, the higher the capacity of the matrix to retain the drug and delay/sustain the release. From the data depicted in Table 3, for matrices containing Carbopol, the higher the concentration of the hydrophilic polymer, the higher the MDT. A direct correlation between MDT and the amount of polymer composing the dosage form has already been reported, regardless of the type of drug and polymer [32], but this trend was not fully confirmed for HPMC-composing tablets, i.e., F-4 has 10% of HPMC and F-3 has 7.5% (Table 7). MDT was higher for F-3 than for F-4 (Table 3).

The accelerated stability studies of F-2 were performed by stressing the formulation under accelerated temperature and relative humidity conditions (40 °C and 75% RH). The results show that it kept the physical properties for three months (Table 4). The content uniformity and friability remained within the tolerance limits set by the European Pharmacopoeia, and no visual signs of changes in color or texture were evident.

**Figure 9:**
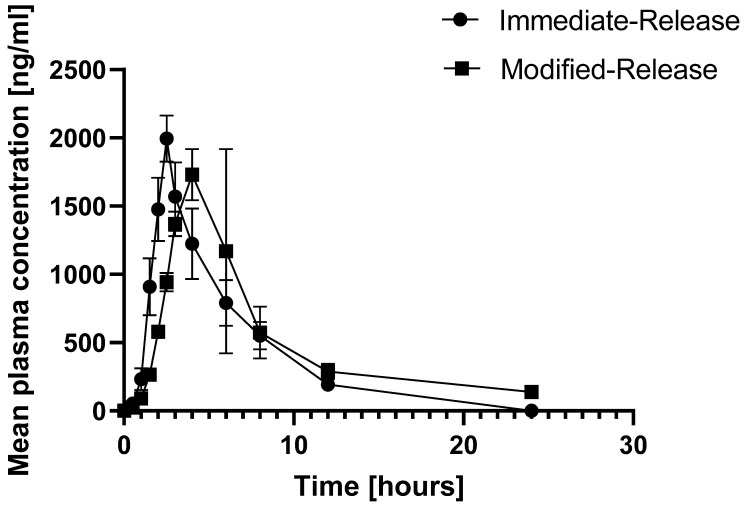
Mean plasma concentrations (ng/mL) recorded over 24 h for acyclovir administered as immediate release formulation versus sustained release formulation.

The relative bioavailability of the developed ACY SR tablets, 400 mg (F-2) given twice daily, was compared with marketed 200 mg ACY tablets given five times daily. The designed SR tablet depicted a plasma concentration-time profile typical for modified-release formulations (Figure 9, Table 5).

**Table 5:** Mean pharmacokinetic profile of acyclovir SR tablets.

Pharmacokinetic Parameters	Type of Formulation	
	Developed Sustained-Release Tablets (F-2)	Immediate-Release Tablets (IR)
AUC_0-t_ ^c^ (ng.h/mL)	11,870.69 ± 1190.02	10,118.54 ± 1151.16
AUC_0-__∞_ ^c^ (ng.h/mL)	13,194.90 ± 1305.39	10,929.35 ± 1249.17
C_max_ ^c^ (ng/mL)	1852.51 ± 78.04	2029.29 ± 104.24
T_max_ ^c^ (h)	5.00 ± 1.10	2.42 ± 0.20
k_el_ ^c^ (h^−1^)	0.11 ± 0.00	0.24 ± 0.01
t_1/2_ ^c^ (h)	6.20 ± 0.12	2.92 ± 0.07

^c^ Results are the mean of six replicates (*n* = 6) ± standard deviation.

The sustained-release F-2 tablets took longer to reach the peak concentration than the immediate-release formulation. They were kept at a higher concentration longer (i.e., lower variation in drug plasma concentration, longer time to reach t_max_). No significant difference in absorption was noted from the outputs obtained for AUC_0-t_. However, the AUC_0-α_ value for sustained-release F-2 tablets was 1.21 times higher than that for immediate-release tablets, indicating more efficient and sustained drug delivery, which would keep the therapeutic plasma ACY levels for a longer time. The exact process was also observed by the lower elimination rate (2.12 times lower for sustained-release F-2 tablets) and higher t_½_ values (2.12 times higher than for the immediate-release tablets). The function of the livers and kidneys was assessed by clinical and biochemical tests and was expected (data not shown).

The pharmacokinetic parameters of the sustained-release F-2 tablets and immediate-release formulation were compared statistically by one-way ANOVA. The pharmacokinetic parameters, including C_max_, t_max_, AUC_0-t_, t_½_, kel, and AUC_0-∞_, of the IR and SR formulations of ACY were significantly different (*p* < 0.05).

## 3. Materials and Methods

### 3.1. Materials

ACY was a gift sample from Matrix Laboratories Ltd. (Hyderabad, India). HPMC (MethocelTMK100CR, apparent viscosity, 2% in water at 20 °C is 80,000–120,000 cP) and Carbopol 910 NF (apparent viscosity, 0.5% in water at 25 °C is 3000–7000 cP) were obtained as gift samples from Noveon (Mumbai, India). Microcrystalline cellulose (Avicel^®^ PH-101) was a gift from Signet Chemical Corporation (Mumbai, India). Polyvinylpyrrolidone (PVP K-30) was a gift sample from Anshul Agencies (Mumbai, India). Magnesium stearate and talc were obtained from S.D. Fine Chemicals (Mumbai, India). All other chemicals were analyzed by high-performance liquid chromatography (HPLC) or analytical grade.

### 3.2. Drug–Polymer Interactions

The chemical interaction between acyclovir (ACY) and selected polymers was evaluated by differential scanning calorimetry (DSC, Netzsch, Yokohama, Japan). DSC thermograms of ACY alone and physical mixtures (1:1 (*w*/*w*)) of drug and hydrophilic polymer (2–4 mg) were heated (50–300 °C) at a constant speed (10 °C/min) in sealed aluminum pans using nitrogen as purging gas and recorded using an empty pan as reference. The device was prepared earlier by the “indium-check” method, which allows checking an instrument’s temperature and heat flow calibration.

### 3.3. Preparation of Polymeric Granules and Tablets

ACY matrix tablets were produced by wet granulation. Firstly, HPMC and Carbopol were sieved using a British Standard Sieve (BSS) of 0.25 mm diameter. The resulting polymers and their combinations were granulated separately, using a solution of PVP K-30 in isopropyl alcohol as a granulation medium. The obtained wet mass was manually passed through BSS with a 1.7 mm diameter, and granules were dried at 60 °C for 3–4 h until a drying loss lower than 2% (*w*/*w*). As a result, the dried granules were passed through BSS 1.0 mm diameter. The compositions of different batches of ACY granules are shown in Table 6. Avicel PH 101, magnesium stearate, and talc were added to the granules to obtain a blend that was compressed on a 10-station rotation tablet machine of 12 mm concave punches (Rimek, Ahmedabad, India). The compression force was kept constant at 9000 N [16]. The composition of the final tablets is given in Table 7. Batches of 100 tablets (400 mg of the drug each tablet) were produced. The drug was added to the powders before wet granulation.

**Table 6:** Composition (in mg) of acyclovir granules.

Code	G-1	G-2	G-3	G-4	G-5	G-6	G-7	G-8	G-9	G-10	G-11	G-12
Acyclovir	400	400	400	400	400	400	400	400	400	400	400	400
HPMC	15	30	45	60	-	-	-	-	7.5	15	22.5	30
Carbopol	-	-	-	-	15	30	45	60	7.5	15	22.5	30
PVP-K-30	30	30	30	30	30	30	30	30	30	30	30	30

**Table 7:** Composition (in mg) of acyclovir tablets.

Code	F-1	F-2	F-3	F-4	F-5	F-6	F-7	F-8	F-9	F-10	F-11	F-12
Granules	G-1	G-2	G-3	G-4	G-5	G-6	G-7	G-8	G-9	G-10	G-11	G-12
Avicel PH101	137	122	107	92	137	122	107	92	137	122	107	92
Mg stearate	12	12	12	12	12	12	12	12	12	12	12	12
Talc	6	6	6	6	6	6	6	6	6	6	6	6
Total weight	600	600	600	600	600	600	600	600	600	600	600	600

### 3.4. Evaluation of Granules

#### 3.4.1. Angle of Repose

The granules were evaluated before compression for their static angle of repose using the funnel method. Accurately weighed samples were placed in the funnel, and its height was adjusted so that the funnel’s tip touched the apex of the granules’ heap. Granules were allowed to free flow through the funnel. Then, the diameter of the obtained powder cone was estimated. In contrast, the angle of repose was determined by applying the following equation [33], where *h* and *r* refer, respectively, to the height of the powder cone and its radius, as in Equation (5):

(5) $$\tan\theta =\frac{h}{r}\;$$

#### 3.4.2. Bulk Density

For the determination of both the loose bulk density (LBD) and tapped bulk density (TBD), 10 mg of granules of each formulation (Table 6) was weighed and firstly lightly shaken to break agglomerates and then placed in a 10 mL graduated test tube. The initial volume (before tapping) was read, and then the graduated test tube was allowed to fall under its weight onto a solid surface from the height of 2.5 cm at a time interval of 2 s. The tapping was continued until no further changes in the volume were noticed. LBD and TBD were determined using the following Equations (6) and (7) [34]:

(6) $$LBD=\frac{Weight\;of\;the\;powder\;\left(g\right)}{Volume\;of\;teh\;packing\;before\;tapping\;\left(\mathrm{mL}\right)}$$

(7) $$TBD=\frac{Weight\;of\;the\;powder\;\left(g\right)}{Volume\;of\;teh\;packing\;after\;tapping\;\left(\mathrm{mL}\right)}$$

#### 3.4.3. Compressibility Index

The Carr’s compressibility index (%) of the granules (Table 6) was calculated as previously described [16] and using the following Equation (8):

(8) $$CI\;\left(\%\right)=\frac{TBD-LBD}{TBD}\times 100$$

#### 3.4.4. Hausner’s Factor (*HF*)

Hausner’s factor (*HF*) translates the interparticle friction [35] and is determined by the ratio between *TBD* and *LBD* to predict powder flow properties, following Equation (9):

(9) $$HF=\frac{TBD}{LBD}$$

### 3.5. Physicochemical Properties of Tablets

#### 3.5.1. Thickness

A digital caliper (Mitutoyo, Kawasaki, Japan) was used to record the thickness of tablets, testing ten tablets taken randomly from each batch and recording the thickness values in mm.

#### 3.5.2. Mass Uniformity Test

The mass uniformity test followed the guidelines set in the European Pharmacopoeia (Ph. Eur.) [22]. Namely, twenty tablets taken randomly of each formulation were weighed using an electronic balance (Sartorius AG, Goettingen, Germany), and values were reported in mg.

#### 3.5.3. Resistance to Crushing Test

The tablet’s resistance to crushing was determined in a hardness tester (Monsanto, Cadmach Machinery, Ahmedabad, India), testing six tablets individually taken randomly from each formulation and ensuring that all fragments of the previous tablet were removed before each testing. The results were recorded in kg/cm^2^ and reported as tablet hardness.

#### 3.5.4. Friability Test

The friability test was run un in a friabilator (Electrolab, Mumbai, India). For each formulation, six tablets taken randomly were weighed before the assay, placed inside the friability, and submitted to 100 rotations for 4 min [21]. The resulting tablets were dedusted and reweighed. The friability was calculated as the percentage of weight loss using the following Equation (10) [16]:

(10) $$Friability\;\left(\%\right)=\frac{Weight_{before\;rotations}-Weight_{after\;rotations}}{Weight_{before\;rotations}}\times 100$$

#### 3.5.5. Content Uniformity Test

The determination of drug content uniformity followed the guidelines in the European Pharmacopoeia (Ph. Eur.) [22]. Ten tablets taken randomly from each formulation were weighed individually, then placed in a mortar and powdered with a pestle. An amount equivalent to 400 mg of ACY (600 mg of powder) was extracted with 100 mL phosphate buffer (pH 7.4) and sonicated over 15 min. The obtained solution was filtered through a Whatman filter paper (0.22 µm pore size, Sigma-Aldrich, Darmstadt, Germany), properly diluted with the same buffer, and the drug content was quantified by HPLC (Waters liquid chromatographic system, Waters Ltd., Mumbai). C18 column was used as a stationary phase. The mobile phase consist of 20 mM disodium hydrogen orthophosphate:acetonitrile (pH 3.0) in the ratio 95:5 (*v*/*v*) at the flow rate of 0.7 mL/min. For satisfactory separation, the detection wavelength was set at 254 nm. As an internal standard, prochlorperazine was used. The retention times of the internal standard and the drugs were 8.9 and 6.2 min, respectively. The linearity for ACY was recorded between 0.25 μg/mL and 2.0 μg/mL. The validation of the chromatographic method was carried out as per ICH guidelines [36].

#### 3.5.6. In Vitro Dissolution and Release Kinetics Assay

The in vitro dissolution assay was performed to evaluate the release profile of the developed sustained-release tablets compared to a standard formulation of the same drug content, as previously described by us [21]. The assays were run in a USP dissolution apparatus (Electrolab, Mumbai, India) under perfect sink conditions. During the first two hours, the dissolution media consisted of 700 mL of 0.1 N HCl (pH 1.2), after which 200 mL of 0.2 M sodium phosphate tribasic solution was added to obtain a final pH of 7.4. Then, 5 mL of each sample was replaced through a 0.45 µm filter by 5 mL of a suitable fresh dissolution medium, which was maintained under the same conditions at preselected intervals up to 24 h. The media were stirred at 100 rpm, and the temperature was maintained at 37 ± 0.5 °C for the experiments. The amount of the drug released was quantified by HPLC as described in Section 3.6.2. Each test was conducted in triplicate, and the results are expressed as mean ± standard deviation (SD). As a result, data were fitted to different kinetic equations, e.g., zero order, first order, and Higuchi. Consequently, the mechanism encountered in releasing ACY from the hydrophilic matrix systems has been described. Graph Pad Software Version 3.05 (Graph Pad, San Diego, CA, USA) was used for calculations.

#### 3.5.7. Stability Studies

The stability of an optimized tablet formulation was studied under accelerated temperature and relative humidity conditions (40 °C and 75% RH). The tablets were packed in screw-capped high-density polyethylene containers and placed in the stability chamber (Thermolab, Mumbai, India) for three months. Sampling was performed every month and submitted to visual inspection of any appreciable changes in the tablet’s surface, thickness, hardness, friability, and drug content, as described above.

### 3.6. Bioavailability Studies

#### 3.6.1. Design of the Study

A randomized bioavailability study was run on six healthy male volunteers (22–25 years old, weighing between 50–70 kg under fasting conditions) to compare the developed sustained-released (SR) optimized tablet formulation (400 mg ACY) with the commercially available immediate-release (IR) tablets (reference product, containing 200 mg of ACY). Following a two-treatment, two-period, two sequence, single-dose, cross-over study, the liver and kidney function of the subjects was evaluated using clinical and biochemical analyses. The volunteers received formulations with 240 mL of water. Blood samples (5 mL) were collected in heparinized centrifuge tubes after dosing at pre-determined time intervals for 24 h. Plasma was separated by centrifugation, and ACY levels in plasma were estimated by a validated reverse phase HPLC method, as described in Section 3.6.2. All of the volunteers signed consent to participate in this study. Moreover, none of them used alcohol or tobacco for at least one week before the survey. The Institutional Review Board has approved the ethics of the study protocol (JSSCP/DPP/013/2009-2010 dated 8 October 2009).

#### 3.6.2. Chromatographic Conditions

Liquid chromatographic separations were achieved using Princeton SPHER (Princeton Chromatography Inc., Cranbury, NJ, USA) C_18_ column (250 mm × 4.6 mm i.d., 5 µm) as the stationary phase. The column and the autosampler tray were kept constant at 40 °C and 4 °C, respectively. A mixture of acetonitrile and 20 mM disodium hydrogen orthophosphate buffer (pH 3.0; 5:95 % (*v*/*v*)) was included in the mobile phase at a flow rate of 0.7 mL/min with an operating pressure of 3000 psi. A Rheodyne-7725i injector (Sigma-Aldrich, Darmstadt, Germany) with a 50 µL loop was used to inject the samples. Detection was carried out at 254 nm using prochlorperazine maleate as an internal standard. The mobile phase was filtered through 0.22 µm membranes and degassed in a Q500 ultrasonicator (Qsonica, VWR International, LLC, Radnor, PA, USA). The experiments were carried out at room temperature (20 ± 2 °C).

#### 3.6.3. Pharmacokinetics

A non-compartment method using PK1 and PK2 solution SimBiology MATLAB^®^ software (MathWorks Inc., Natick, MA, USA) was used to determine the pharmacokinetic parameters of ACY. The maximum plasma concentration (C_max_) and the time at which maximum plasma concentration (t_max_) was recorded were obtained directly from the concentration-time curve. The elimination rate constant (k_el_) was determined by applying a log-linear regression analysis to at least the last three concentrations of ACY. Both areas were under the plasma concentration-time curve from zero to the last measurable time point (AUC_0-t_) and the one under the plasma concentration-time curve up to the infinity time point (AUC_0-α_) determined by applying the linear trapezoidal method. Half-life (t_½_) was calculated as 0.693/k_el_ [37].

### 3.7. Statistical Analysis

GraphPad Prism software (Intuitive Software for Science, San Diego, CA, USA) was used to perform statistical analysis. Relevant pharmacokinetic parameters, i.e., C_max_, t_max_, t_½_, k_el_, AUC_0-t_, and AUC_0-α_, are given as the mean ± standard deviation (SD). A one-way ANOVA was employed in the statistical analysis with significance defined as *p* < 0.05. Relative SD is expressed in percent and is obtained by multiplying the standard deviation by 100 and dividing the result by the average.

## 4. Conclusions

In this work, we produced stable tablets composed of hydrophilic polymers by wet granulation for the biomedical administration of acyclovir. The optimal HPMC-based formulation submitted to accelerated stability studies was stable for three months. Moreover, HPMC-composed tablets were superior in modified drug release properties compared to carbomer- and HPMC/carbomer-based tablets. As a result, oral tablets kept their mechanical/physical properties under stress conditions. The production method ensured mass and content uniformity of the sustained-release tablets, found in vivo to deliver acyclovir through oral administration, showing an extent of absorption higher than the immediate-release counterparts. The highest plasma concentration was reached later than the immediate-release formulation but remained longer within the therapeutic window, anticipating a longer therapeutic effect with reduced side effects. This study has proven the extent of drug absorption from the sustained release tablets was significantly higher than that from immediate-release pills. This may improve the drug’s antiviral properties connected with the lower elimination rate and enhanced acyclovir half-life.

## Figures and Tables

**Table 1:** Physical properties of granules obtained from different formulations (*n* = 3).

Code	Angle of Repose (°)	Tapped Bulk Density (gm/cm^3^)	Loose Bulk Density (gm/cm^3^)	Carr’s Index (%)	Hausner’s Factor
G-1	23.05 ± 0.05	0.40 ± 0.05	0.33 ± 0.04	16.68 ± 0.08	1.200
G-2	23.34 ± 0.06	0.38 ± 0.04	0.32 ± 0.07	16.12 ± 0.05	1.192
G-3	22.38 ± 0.09	0.42 ± 0.09	0.33 ± 0.03	20.01 ± 0.07	1.250
G-4	22.79 ± 0.04	0.43 ± 0.05	0.32 ± 0.02	25.80 ± 0.05	1.348
G-5	23.05 ± 0.06	0.42 ± 0.03	0.32 ± 0.05	22.58 ± 0.08	1.292
G-6	23.58 ± 0.03	0.38 ± 0.06	0.33 ± 0.05	13.34 ± 0.07	1.154
G-7	22.01 ± 0.07	0.40 ± 0.04	0.34 ± 0.06	13.80 ± 0.06	1.160
G-8	22.55 ± 0.05	0.42 ± 0.06	0.32 ± 0.04	22.58 ± 0.04	1.292
G-9	23.70 ± 0.06	0.43 ± 0.05	0.33 ± 0.04	23.34 ± 0.09	1.305
G-10	23.05 ± 0.04	0.40 ± 0.09	0.33 ± 0.06	16.68 ± 0.08	1.200
G-11	23.09 ± 0.03	0.40 ± 0.08	0.32 ± 0.06	19.35 ± 0.06	1.240
G-12	21.99 ± 0.05	0.43 ± 0.07	0.32 ± 0.07	25.80 ± 0.05	1.348

**Table 2:** Physical properties of modified-release tablets containing acyclovir.

Code	Mass Uniformity (g) *n* = 20	Hardness (kg/cm^3^) *n* = 6	Friability (%) *n* = 6	Thickness (mm) *n* = 10	Drug Content Uniformity (%) *n* = 5
F-1	0.6036 ± 0.006	5.50 ± 0.30	0.412 ± 0.009	5.21 ± 0.04	96.22 ± 1.4
F-2	0.6017 ± 0.009	5.68 ± 0.19	0.472 ± 0.015	5.23 ± 0.10	97.87 ± 1.6
F-3	0.6022 ± 0.005	5.62 ± 0.22	0.419 ± 0.017	5.22 ± 0.07	96.45 ± 1.5
F-4	0.6010 ± 0.007	5.59 ± 0.16	0.422 ± 0.018	5.25 ± 0.06	98.21 ± 1.9
F-5	0.5997 ± 0.004	5.48 ± 0.15	0.466 ± 0.010	5.30 ± 0.08	97.68 ± 1.3
F-6	0.5992 ± 0.007	5.16 ± 0.22	0.484 ± 0.012	5.29 ± 0.05	98.33 ± 1.2
F-7	0.6011 ± 0.006	5.82 ± 0.47	0.492 ± 0.015	5.27 ± 0.07	97.66 ± 1.1
F-8	0.6032 ± 0.008	5.77 ± 0.27	0.477 ± 0.017	5.26 ± 0.09	97.54 ± 1.9
F-9	0.6041 ± 0.010	5.71 ± 0.33	0.480 ± 0.019	5.28 ± 0.08	97.22 ± 1.6
F-10	0.6020 ± 0.012	5.75 ± 0.39	0.460 ± 0.027	5.27 ± 0.04	98.94 ± 1.8
F-11	0.6031 ± 0.009	5.66 ± 0.37	0.450 ± 0.022	5.29 ± 0.05	98.11 ± 1.3
F-12	0.6038 ± 0.008	5.54 ± 0.26	0.470 ± 0.030	5.30 ± 0.05	98.17 ± 1.7

*n* is the number of measurements.

**Table 3:** Regression coefficient (r^2^) values obtained from different kinetic models, diffusion exponent (*n*) of Peppas model, and mean dissolution time (MDT) of acyclovir released from developed tablets.

Code	Zero Order (r^2^)	First Order (r^2^)	Higuchi (r^2^)	Peppas		MDT (Hours)
				n	(r^2^)	
F-1	0.9468	0.9600	0.9901	0.5994	0.9852	0.245
F-2	0.8777	0.9400	0.9412	0.5011	0.9799	1.820
F-3	0.9542	0.9950	0.9897	0.5112	0.9892	2.300
F-4	0.9264	0.9911	0.9838	0.5084	0.9901	2.080
F-5	0.8653	0.9487	0.9738	0.1048	0.9571	0.231
F-6	0.8602	0.9584	0.9732	0.1571	0.9728	0.291
F-7	0.8652	0.9829	0.9715	0.2300	0.9714	0.352
F-8	0.8378	0.9939	0.9545	0.3305	0.9538	0.487
F-9	0.9167	0.9010	0.9940	0.1110	0.9929	0.231
F-10	0.8716	0.9516	0.9774	0.1645	0.9733	0.300
F-11	0.8554	0.9758	0.9754	0.2249	0.9644	0.350
F-12	0.8185	0.9978	0.9484	0.2835	0.9558	0.469

**Table 4:** Stability data at the end of three months for acyclovir SR tablets.

Parameter	Initial ^a^	Real Time ^a^	Accelerated ^a^
Thickness (mm); (*n* = 10)	5.28 ± 0.11	5.27 ± 0.12	5.29 ± 0.14
Hardness (kg/cm^2^); (*n* = 6)	5.30 ± 0.05	5.29 ± 0.10	5.29 ± 0.20
Friability (%); (*n* = 6)	0.45 ± 0.09	0.44 ± 0.08	0.47 ± 0.09
Drug content (%); (*n* = 5)	99.64 ± 1.10	97.84 ± 1.90	98.41 ± 1.60

^a^ Results represent the mean of replicate determination with the standard deviation.

## Data Availability

Not applicable.

## References

[1] O’Brien J.J., Campoli-Richards D.M. (1989). Acyclovir. An updated review of its antiviral activity, pharmacokinetic properties and therapeutic efficacy. Drugs.

[2] Arnal J., Gonzalez-Alvarez I., Bermejo M., Amidon G.L., Junginger H.E., Kopp S., Midha K.K., Shah V.P., Stavchansky S., Dressman J.B. (2008). Biowaiver monographs for immediate release solid oral dosage forms: Aciclovir. J. Pharm. Sci..

[3] Kristl A., Srčič S., Vrečer F., Šuštar B., Vojnovic D. (1996). Polymorphism and pseudopolymorphism: Influencing the dissolution properties of the guanine derivative acyclovir. Int. J. Pharm..

[4] Wagstaff A.J., Faulds D., Goa K.L. (1994). Aciclovir. A reappraisal of its antiviral activity, pharmacokinetic properties and therapeutic efficacy. Drugs.

[5] Vergin H., Kikuta C., Mascher H., Metz R. (1995). Pharmacokinetics and bioavailability of different formulations of aciclovir. Arzneimittelforschung.

[6] de Miranda P., Blum M.R. (1983). Pharmacokinetics of acyclovir after intravenous and oral administration. J. Antimicrob. Chemother..

[7] Hosny E.A., al-Helw A.R., al-Dardiri M.A. (1997). Comparative study of in-vitro release and bioavailability of sustained release diclofenac sodium from certain hydrophilic polymers and commercial tablets in beagle dogs. Pharm. Acta Helv..

[8] El-Sayed Y.M., Niazy E.M., Khidr S.H. (1995). In vivo evaluation of sustained release microspheres of metoclopramide hydrochloride in beagle dogs. Int. J. Pharm..

[9] Shin S., Kim T.H., Jeong S.W., Chung S.E., Lee D.Y., Kim D.-H., Shin B.S. (2019). Development of a gastroretentive delivery system for acyclovir by 3D printing technology and its in vivo pharmacokinetic evaluation in Beagle dogs. PLoS ONE.

[10] Fuertes I., Miranda A., Millán M., Caraballo I. (2006). Estimation of the percolation thresholds in acyclovir hydrophilic matrix tablets. Eur. J. Pharm. Biopharm..

[11] Garg R., Gupta G. (2007). Development and evaluation of gastro retentive floating drug delivery system for acyclovir. Asian J. Pharm..

[12] Lordi G.N., Lachman L., Lieberman A.H., Kanig L.J. (1987). Sustained release dosage forms. The Theory and Practice of Industrial Pharmacy.

[13] Melia C.D. (1991). Hydrophilic matrix sustained release systems based on polysaccharide carriers. Crit. Rev. Ther. Drug Carrier Syst..

[14] Alderman D.A. (1984). A review of cellulose ethers in hydrophilic matrices for oral controlled release dosage forms. Int. J. Pharm. Tech. Prod..

[15] Gupta B., Mishra V., Gharat S., Momin M., Omri A. (2021). Cellulosic Polymers for Enhancing Drug Bioavailability in Ocular Drug Delivery Systems. Pharmaceuticals.

[16] Nagasamy Venkatesh D., Meyyanathan S.N., Shanmugam R., Kamatham S.S., Campos J.R., Dias-Ferreira J., Sanchez-Lopez E., Cardoso J.C., Severino P., Souto E.B. (2021). Physicochemical, pharmacokinetic, and pharmacodynamic characterization of isradipine tablets for controlled release. Pharm. Dev. Technol..

[17] Sweetman S.C. (2004). Martindale: The Complete Drug Reference.

[18] Kurakula M., Rao G.S.N.K. (2020). Pharmaceutical assessment of polyvinylpyrrolidone (PVP): As excipient from conventional to controlled delivery systems with a spotlight on COVID-19 inhibition. J. Drug Deliv. Sci. Technol..

[19] Viera-Herrera C., Santamaría-Aguirre J., Vizuete K., Debut A., Whitehead D.C., Alexis F. (2020). Microcrystalline Cellulose Extracted from Native Plants as an Excipient for Solid Dosage Formulations in Drug Delivery. Nanomaterials.

[20] Halder S., Islam A., Shuma M.L., Bachar S.C. (2014). Development and evaluation of Water dispersible tablets of Acyclovir. Int. J. Adv. Res. Biol. Sci..

[21] Venkatesh D.N., Meyyanathan S.N., Shanmugam R., Zielinska A., Campos J.R., Ferreira J.D., Souto E.B. (2020). Development, in vitro release and in vivo bioavailability of sustained release nateglinide tablets. J. Drug Deliv. Sci. Technol..

[22] (2019). European Pharmacopoeia.

[23] Nagasamy Venkatesh D., Sankar S., Meyyanathan S.N., Elango K., Suresh B., Santhi K. (2010). Design and Development of Prochlorperazine Maleate Sustained Release Tablets: Influence of Hydrophilic Polymers on the Release rate and In vitro Evaluation. Int. J. Pharm. Sci. Nanotechnol..

[24] Meyyanathan S.N., Rajan S., Muralidharan S., Mahesh Kumar S., Krishnaraj K., Suresh B. (2008). Formulation and Evaluation of Dextromethorphan Hydrobromide Sustained Release Tablets. Drug Deliv..

[25] Higuchi T. (1963). Mechanism of sustained-action medication. Theoretical analysis of rate of release of solid drugs dispersed in solid matrices. J. Pharm. Sci..

[26] Paul D.R. (2011). Elaborations on the Higuchi model for drug delivery. Int. J. Pharm..

[27] Korsmeyer R.W., Gurny R., Doelker E., Buri P., Peppas N.A. (1983). Mechanism of solute release from porous hydrophilic polymers. Int. J. Pharm..

[28] Peppas N.A. (1985). Analysis of Fickian and non-Fickian drug release from polymers. Pharm. Acta Helv..

[29] Peppas N.A., Sahlin J.J. (1989). A simple equation for the description of solute release. III. Coupling of diffusion and relaxation. Int. J. Pharm..

[30] Ritger P.L., Peppas N.A. (1987). A simple equation for description of solute release I. Fickian and non-fickian release from non-swellable devices in the form of slabs, spheres, cylinders or discs. J. Control. Release.

[31] Wadher K.J., Kakde R.B., Umekar M.J. (2011). Study on sustained-release metformin hydrochloride from matrix tablet: Influence of hydrophilic polymers and in vitro evaluation. Int. J. Pharm. Investig..

[32] Reza M.S., Quadir M.A., Haider S.S. (2003). Comparative evaluation of plastic, hydrophobic and hydrophilic polymers as matrices for controlled-release drug delivery. J. Pharm. Pharm. Sci..

[33] Travers D.N., Carter J.S. (1986). Powder flow and compaction. Cooper and Gunn’s Tutorial Pharmacy.

[34] Shah D., Shah Y., Pradhan R. (1997). Development and Evaluation of Controlled-Release Diltiazem HCl Microparticles Using Cross-Linked Poly(Vinyl Alcohol). Drug Dev. Ind. Pharm..

[35] Lachman L., Liberman H.A., Kanig J.L. (1987). The Theory and Practice of Industrial Pharmacy.

[36] ICH Q2A (1994). Harmonised Tripartite Guideline: Text on Validation of Analytical Procedures.

[37] Ritschel W.A. (1992). Handbook of Basic Pharmacokinetics including Clinical Applications.

